# DSMs and the Brazilian psychiatric reform

**DOI:** 10.3389/fpsyg.2015.00401

**Published:** 2015-04-13

**Authors:** Fuad Kyrillos Neto, Jacqueline de Oliveira Moreira, Christian I. L. Dunker

**Affiliations:** ^1^Research and Extension Center, Department of Psychology, Federal University of São João del-ReiSão João Del Rei, Brazil; ^2^Extended General Practice in Health, Department of Psychology, Pontifical Catholic University of Minas GeraisBelo Horizonte, Brazil; ^3^Laboratory of Social Theory, Philosophy and Psychoanalysis, Department of Clinical Psychology, Institute of Psychology, University of São PauloSão Paulo, Brazil

**Keywords:** psicopatologia, diagnosis, DMS, psychiatric reform, ways of living

The present text proposes a reflection on the point of recent history of changes in the Brazilian public policies for mental health in which DSM's (*Diagnostic and Statistic Manual of Mental Disorders*) practical psychopathology was introduced in services inspired by psychiatric reform.

The counterpoint of these considerations is psychoanalytic clinic, which focuses on “spontaneously achieved diagnosis”—which means that the person who is being submitted to analysis is able to name and to understand their own condition. Psychoanalytic clinic is based on free association, on transference and on discourse; these being the principles which make it possible for the subject to build knowledge on their own symptoms throughout the treatment. This enables us to think that the ability to name and understand their own suffering can be achieved by forms of life which have productive naming practices (self-diagnosis). We believe that using DSM and insisting in the movement of reinsertion (often forced) may produce the effect of silencing the individual and diminishing the possibilities of clinically listening to singular experience.

In this manuscript what interests the most are the impacts of using DSM's classification combined with a discourse which privileges citizenship, “unmedicalization” and social acts in the movement against madhouses.

This movement, which began in Brazil in the 1980s, can be related to major social and ideological changes that took place in the end of Brazilian Military Dictatorship. These changes reached many different sectors of society, including mental health services, which were drastically modified in regards to the way they are organized and how they understand and offer mental health care. In the beginning of the aforementioned decade, there were two main forms of mental health care: the public mental hospitals (where patients actually spent the rest of their lives) and a rapidly growing chain of private psychiatric clinics (Delgado, 2008). In that period, marked by the democratization of Brazilian political system and by the consequent public acceptance of political-ideological debate, some previously marginalized psychiatric and psychoanalytical perspectives found an opportunity to be broadcasted, and, thus, show alternatives for the treatment of psychological phenomena.

The Brazilian Movement of Mental Health Professionals proposed, in that context, a critical review of the hegemonic and centralizing role of psychiatric hospitals as well as of mental health services. According to this new proposition, financial resources and the forms of care and treatment should invariably preserve personal dignity and human and civil rights as well as provide ways of maintaining the patient in their own community, since isolation and social disconnection were considered the main problems of interning patients in mental institutions. Brazilian legislation should adapt in order to ensure that mental patients would have their human and civil rights respected, to reorganize community mental health services and to make sure these new configurations are taken into practice. Furthermore, according to the proposals of this movement, human resources in mental health and psychiatry should be trained in accordance to a model in which community service prevailed and the new principles for hospitalization were to be followed.

In order to make these directives possible, it is necessary to organize replacement services, which must be prepared to take on new tasks and to respond to the necessities of patients and their families. The Centers of Psychosocial Care (*Centros de Atenção Psicossociais*—CAPS) certainly constitute the most creative and advanced alternative for reaching these goals. There, a contract is established with the patient, regarding the type of treatment, the number of times the patient will go to the hospital every week and the way they will deal with the medication (either at home or at CAPS).

Today, the CAPS are ruled by Ordinance^1^ no. 336/GM, published on February 19, 2002, and integrate the Unified Health System (*Sistema Único de Saúde*—SUS). This Ordinance recognized the Centers and amplified CAPS's functioning and complexity, defining that these services must provide continuous treatment to people who suffer with severe and persistent mental illnesses in a certain territory. They must also provide clinical care and psychosocial rehabilitation, substituting, thus, the hospital-centered model which prevailed in the past. CAPS must also make an effort to avoid hospitalization and promote citizenship and social inclusion of patients and their families.

The Centers which are open 24 h a day provide rear beds for both male and female patients, who have access to many diverse types of care offered by the service: day and night hospitality; day-time hospital; home visits; and access to psychiatric medication. In case of a more critical state, indicating the necessity of a more intense accompaniment, the patient will remain in the center (day and night hospitality) and will use one of the rear beds.

Nicácio (1994) points out five essential characteristics of therapeutic practices carried out by these services, namely: guaranteed rights to asylum in the center (which does not translate into isolation or exclusion); rapid responses to critical situations; insertion in their community; investment inversion (meaning to emphasize social production of patients, i.e., without worrying about clinical structure or psychopathologic outlines); and, finally, the process of social valorization, comprehended as the active institutional participation in the process of social interchange.

When analyzing these five characteristics, defined as essential for the ideological project which guide the functioning of the service, the absence of a clinical dimension is noticed. The “investment inversion” to which referred Nicácio (1994) proposes that psychiatry should not emphasize pathology; instead, it should contemplate the complex existence of these patients and their insertion in social context. Therefore, there has been an abdication of the clinical point of view in favor of social context.

In DSM's case, one can raise the hypothesis that this characterization proposal is inspired by pragmatism, renouncing, thus, the notion of mental illness in favor of the idea of a disorder: something that is not in accordance to the previously established order. Therefore, DSM is organized in order to find trustworthy, temporary and operational categories which allow the overcoming of terminological misunderstandings in the field of Psychopathology (Pereira, 2009).

In regards to the use of DSM in public policies concerning mental health, it can be stated that, motivated by the desire to organize an efficient form of investment in public health policies (including policies for mental health care), the managers must know which are the most frequent and prevailing clinical entities in a certain community as well as the real efficacy of the several available therapeutic possibilities. According to Pereira (1996), this is a perspective that thinks of medicine as a concrete form of intervention in the order of life, as well as in social institutions. Mental suffering, in this perspective, is seen as a matter of public health^2^. In contemporaneity, defining clinical practice through efficacy, based in rapid results, becomes an ethical ideal.

It is important to remember that, since the first version of DSM, in 1952, there can be noticed a movement of revitalization of several diagnostic classes which subdivide psychodynamic classes, such as neurosis and psychosis, into increasingly smaller symptomatic units. Thus, it becomes more and more common to see all sorts of people recognizing themselves in a group of clinical signs with some diagnostic value. It appears that this type of diagnosis finds itself in consonance with the globalization of capital, since there is a globalization of presentation and cataloging of the different ways to suffer.

Lately, the conflict between the possible diagnostic reasons and their implications in treatment and organization of services has been intensified. A response to this intensification, a third version of DSM has been developed, and it represents a turning-point in the relations between Psychiatry and Psychoanalysis, since it proposes a non-theoretical classificatory system, operational in great psychiatric syndromes, which can modify the concept of research and psychiatric practice (Mayes and Horwitz, 2005). This conducts us to a situation in which psychic phenomena are comprehended and defined through their topography, and the definition of a mental disorder happens based on the simultaneous manifestation of several symptoms. The subject and their life-story become dispensable for treatment choices.

Therefore, we argue that, even though they have given positive contributions to the thought regarding psychological treatments, DSM and the practices which insist in social re-insertion disconnected of any subjective consideration may lose sight of the individual and their story.

## Conflict of interest statement

The authors declare that the research was conducted in the absence of any commercial or financial relationships that could be construed as a potential conflict of interest.

## Footnotes

^1^BRASIL. Portaria n 336/GM, de 19 de fevereiro de 2002. Available online at: http://dtr2004.saude.gov.br/susdeaz/legislacao/arquivo/39_Portaria_336_de_19_02_2002.pdf [Accessed 02 February 2015].

^2^Foucault defines Liberalism as the frame of biopolitics. His analysis emphasizes the paradoxal role of society in regards to the government: society tends to limit itself, but the government also intervenes in society in order to produce, guarantee and multiply the liberty necessary for Economic Liberalism (Foucault, 2008).
